# 3D skin models in domestic animals

**DOI:** 10.1186/s13567-020-00888-5

**Published:** 2021-02-15

**Authors:** Laurent Souci, Caroline Denesvre

**Affiliations:** ISP, INRAE, Université de Tours, Equipe BioVA, Centre Val de Loire, 37380 Nouzilly, France

**Keywords:** Skin, Explant, Skin equivalent, Reconstructed epidermis, Stem cells, Keratinocyte, Mammals, Birds

## Abstract

The skin is a passive and active barrier which protects the body from the environment. Its health is essential for the accomplishment of this role. Since several decades, the skin has aroused a strong interest in various fields (for e.g. cell biology, medicine, toxicology, cosmetology, and pharmacology). In contrast to other organs, 3D models were mostly and directly elaborated in humans due to its architectural simplicity and easy accessibility. The development of these models benefited from the societal pressure to reduce animal experiments. In this review, we first describe human and mouse skin structure and the major differences with other mammals and birds. Next, we describe the different 3D human skin models and their main applications. Finally, we review the available models for domestic animals and discuss the current and potential applications.

Most three-dimensional (3D) skin models have been developed in humans and were subsequently adapted to animals. This is mainly due to an easy accessibility to human skin specimens, its flat architecture, an active research for burn repairs, and the wide range of applications: e.g., pharmacology, modelling of skin diseases, and cosmetology. In addition, the strong societal pressure to reduce the use of animals for research purposes since the 1990s boosted the establishment of reliable reconstructed skin models as alternatives. Indeed, these novel 3D skin models made possible the ban on cosmetic testing on animals in the European Union in 2013 and in some other countries afterward. The first part of this review briefly describes the skin structure, functions, and skin stem cells. The second part describes the main methodologies of 3D skin production in humans since the last seventy years. The third part reviews the 3D skin models developed in livestock, poultry, and pets. As only few of these 3D skin models are currently available, we focus on potential future veterinary applications as a justification for the development of such 3D skin models. In our opinion, implementing 3D reconstructed skin models for poultry will be key to understand the role of skin differentiation in Marek disease virus biology.

## Skin, a complex organ most dedicated to body protection and rich in stem cells

### Structure, origin and functions

The skin is a flat organ covering the entire body and one of the largest in surface (about 2 m^2^ and 6 to 10 kg for an adult human) [1]. Skin has multiple functions essential to life, the major one being a barrier to protect the body from the environment. The skin limits (i) injuries and penetration of inert elements (chemicals, physical particles), microorganisms (protozoa, worms, bacteria, fungi, and viruses) and small insects, (ii) water loss of the body, and (iii) DNA damage induced by solar ultraviolet radiations. Additionally, the skin has sensory (nerves, vibrissae), thermoregulation (sweat gland, hair and feathers, and blood flow), and immune (Langerhans cells) functions. Moreover, skin appendages contribute to communication (i.e., cat with erect hairs, courtship display in birds) and to locomotion (flight feathers of birds, horse hoof).

**Figure 1:**
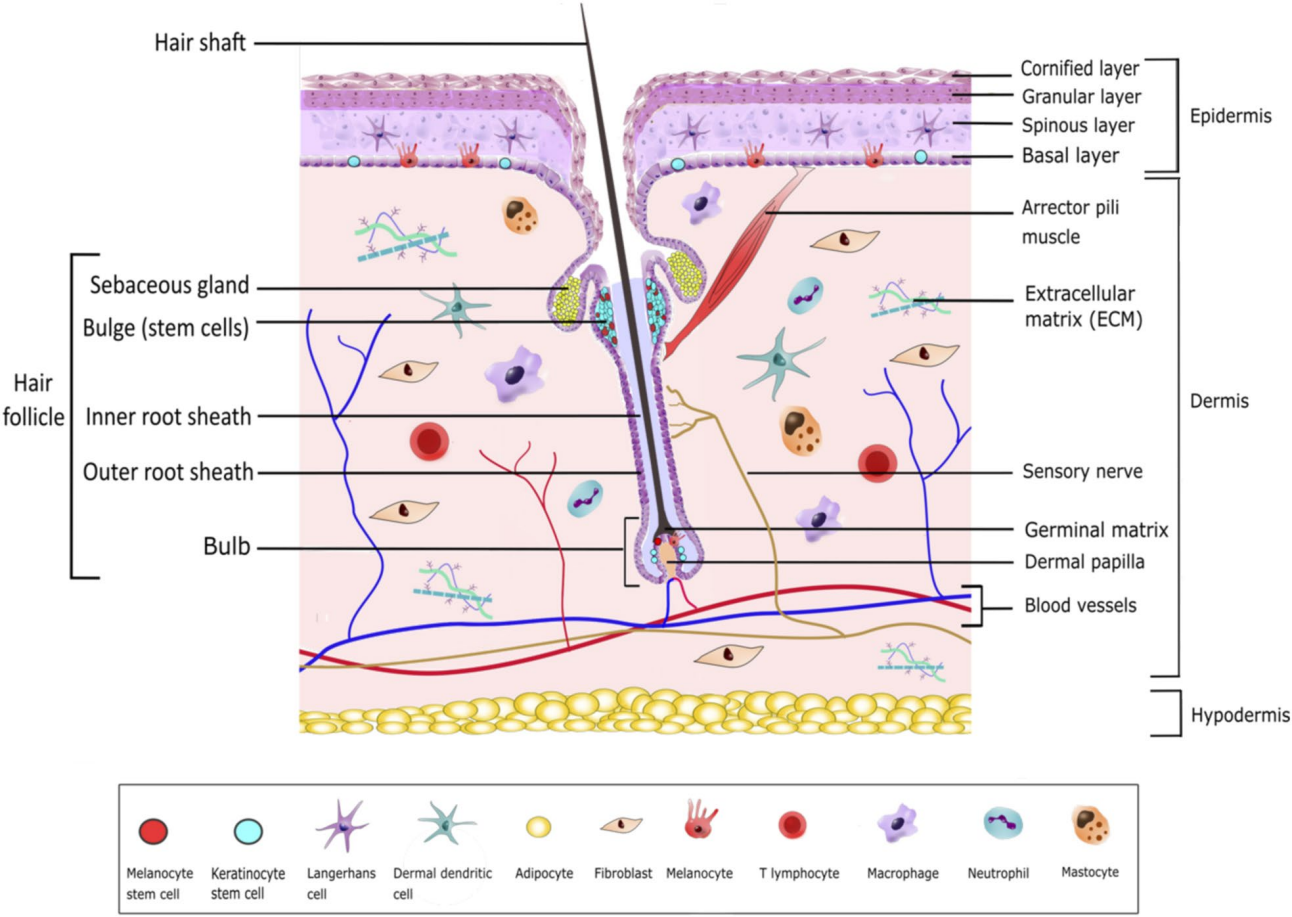
**Overall structure of the mammalian skin with hair follicle. Hypodermis, dermis, and epidermis constitute the skin.** The epidermis is a specialized epithelium, mostly composed of keratinocytes at different stages of differentiation (see text). The dermis, located below the epidermis, consists of a fibrous scaffold composed of ECM, fibroblasts, and immune cells. The dermis hosts blood vessels, nerves, and hair follicles. The bulge, located in the outer root sheath of the hair follicle, houses stem cells which constantly replenish the hair follicle with new cells (keratinocytes, melanocytes), and replenish the sebaceous gland and the epidermis after injuries.

The skin is constituted of three overlaid parts: the hypodermis, the dermis, and the epidermis, the latter being the outermost layer in contact with the air (Figure 1). The hypodermis also called the subcutaneous tissue, mostly formed of lipid cells, has a role as protective padding, insulation, and energy reservoir. The dermis consists of an extracellular matrix (ECM; fibers and glycosaminoglycans) and cells, mostly fibroblasts and immune cells. Fibroblasts deposit the collagen and elastic fibers that give the skin its elasticity. The dermis harbors blood vessels, nerves, glands, and skin appendages (e.g., hair in the hair follicle and the base of nails and claws). The complex structure of a hair follicle has led to consider it as a mini-organ. Glands and skin appendages will not be reviewed in detail in this article because most skin models are currently devoid of these complex structures. Indeed, current skin models are either constituted of an epidermis alone, or an epidermis associated with a dermis. Most of the thickness of the skin is formed by the dermis which is connected to the epidermis by a basal membrane. This basal membrane is composed of ECM proteins, and serves as a “proliferation-promoting platform” for the epidermis [2]. The epidermis is a stratified squamous epithelium mostly composed of keratinocytes (95%). In mammals, these specialized epithelial cells are organized in four layers (from the most internal to the most external layer): the basal (or stratum germinativum), spinous (or stratum spinosum), granular (or stratum granulosum), and cornified (or stratum corneum) layers. Each layer contains keratinocytes at different stages of differentiation, from basal keratinocytes in the basal layer to corneocytes in the cornified layer. Basal keratinocytes are the only proliferative keratinocytes. At their ultimate stage of differentiation, keratinocytes become corneocytes. These cells are enucleated dead cells, resulting from a cell-death program referred to as cornification [3], during which they lose a large amount of water. Corneocytes are constantly shedding, a process called desquamation, and renewed by cells arising from underneath. In normal human skin, the complete differentiation of a keratinocyte and its progression from the basal layer toward the surface takes 40 to 56 days [4], depending on the individual’s age (it is quicker in children and young adults). The age of the donors could therefore influence the quality of the skin used in in vitro models. In mouse, this differentiation process is of only 8 to 10 days [5]. In non-pathologic skin, the other 5% of epidermal cells are melanocytes, Merkel cells, and Langerhans cells. Melanocytes produce melanin which absorbs UV and give different colors to skin as well as hair or feathers [6, 7]. The number of each cell type per mm^2^ of skin and the ratios of different cell types in the human epidermis have been empirically estimated (reviewed in [8]). For example, the ratio of melanocytes or Langerhans cells to nucleated epidermal cells is of 1:36 and 1:53, respectively. This information is important for full skin reconstitution in vitro.

In a healthy skin, various immune cells have also been identified: antigen presenting cells [Langerhans cells, several subsets of dendritic cells (DCs), notably dermal DCs, and macrophages], and resident T cells [including regulatory T cells (Treg)], monocytes, mast cells, and neutrophils [9–11]. Langerhans cells are located in the epidermis, whereas the other immune cells are found in the dermis. Interestingly, immune cells in the skin maintain a subtle balance between a tolerogenic and an immunogenic state through innate and acquired immune response [12]. They can either detect invasive pathogens and induce a protective host response or maintain a tolerant environment towards foreign antigens. Keratinocytes, which produce Toll-like receptors, cytokines, chemokines, and growth factors can also orientate the immune response [9, 12]. In mammals, anti-microbial peptides (i.e. ß-defensins) as well as a variety of lipids present at the surface of the skin assist with microorganisms defense.

Healthy skin hosts various microorganisms on its surface, the skin microbiome. The composition and function of the skin microbiome has been studied in humans [13], as well as in dogs and chickens [14, 15]. Interestingly, the skin microbiota differs from the gut microbiota in that the absence of skin microbiota (e.g., in germ-free mice) appears not deleterious for skin development and morphology [16], while the absence of gut microbiota negatively affects gut development.

The morphological aspect and thickness of the skin varies according to clades, species, race, and even genders, age, and body location. In humans, the skin is thick up to several mm over the sole of the feet in adults. In domestic mammals, the haired skin is thickest over the dorsal surface of the body compared to ventral region [17]. The epidermis is naturally thicker in surfaces that need enhanced protection [17]. Similarly, the stratum spinosum of dogs’ footpads may contain up to 20 layers whereas it is made of only 1 or 2 layers in haired skin [17].

The skin components are coming of different embryological origins: the dermal fibroblasts originate from the mesoderm and the neural crest while the epidermal keratinocytes originate from the ectoderm [18]. Melanocytes are derived from the neural crest [19]. Langerhans cells originate from a subpopulation of bone-marrow CD34+-derived cells expressing the skin homing receptor cutaneous lymphocyte antigen (CLA) [20]. Langerhans cells may persist by local-self renewal [21]. Epidermal structures, such as different types of glands (i.e., sweet glands, sebaceous glands) and skin appendages (i.e., hair, feathers, nails, claws), form via cell–cell interactions with dermal fibroblasts [18, 22].

### Major skin peculiarities in mammals and birds

The overall skin structure is well conserved across different mammalian species and body sites [2], although in the interfollicular epidermis, the number of stacked cells in a layer can vary according to the species. For example, the stratum spinosum has 1 or 2 cell layers in the haired-skin regions of mice, dogs, and cats, and up to 4 in humans, as well as in large animals [17]. The main differences between humans and most domestic and laboratory mammals concern the appendages: hair forming a fur, feathers a plumage, presence of vibrissa with tactile function in some domestic animals (e.g., cats and dogs). Hair and feathers are constantly shed and renewed, according to a cycle. The different phases of hair and feather follicle cycles are variable according to the species. The duration of the growth phase (or anagen) which is 4 to 7 years for the hair of human scalp or human male beard, is only of a few weeks in mouse.

Birds skin presents several differences when compared to mammals’ skin. First, the skin is usually thinner and accommodates feather follicles, the homologues of hair follicles. The main difference between avian and mammalian epidermis is the absence of a granular layer involving filaggrin in birds [23]. Another characteristic of avian keratinocytes is the presence of lipid droplets in their cytoplasm, absent in mammals’ keratinocytes [24, 25]. Importantly, numerous keratinocyte markers described in mammals (e.g., like K5/K14 keratins pair and involucrin) are conserved [25, 26]. In contrast to mammals, melanocytes usually inhabit only the feather follicles and not the interfollicular epidermis in birds [27]. The silky chicken is an exception, with melanocytes populating also the dermis and connective tissues [27, 28]. Birds have a unique sebaceous gland, named uropygial, located dorsally at the base of the tail [28], while mammals have a hair follicle associated with two sebaceous glands, which are required for the emergence of the hair shaft [29]. Lastly, besides feathers, birds show two major hard skin appendages, the scales of the legs and feet, and the beak [28]. Cysteine rich proteins arranged in pleated sheets named corneous beta-proteins (CBP) constitute hard appendages of birds and reptiles (for review see [30]) and have no orthologues in mammals, of which the hair shaft and claws are formed by a special set of keratins [31].

### Skin stem cells

Due to its composition, its constant renewal and the need of repair after wounds, the skin hosts different types of stem cells (or progenitors): tissular and multipotent stem cells*.* Stem cells are located in interfollicular skin and mostly in hair or feather follicles [32–34].

Interfollicular epithelial stem cells reside within the basal layer in mammals [33]. Two types of basal keratinocytes have been described: stem cells and transit amplifying keratinocytes, the second displaying a limited capacity of division [35]. These self-renewing cells adhere to the basal membrane through integrins/laminin interactions. Integrins are considered as good markers for these cells. Factors secreted by dermal fibroblasts (such as insulin-like-growth factor, fibroblast growth factor 7, fibroblast growth factor 10, and epidermal growth factor receptor ligands) promote basal keratinocytes’ proliferation [34]. Basal keratinocytes express keratin 5 and keratin 14 [33]. When basal keratinocytes detach from the basal membrane and move upward, a process called delamination, they lose their proliferating property and start to differentiate and express keratin 1 and keratin 10. Basal keratinocytes isolated from the epidermis are able to propagate in vitro for several generations [36]. In fact, only 10% of human basal keratinocytes from the interfollicular epidermis and the hairless skin (soles) are stem cells and able to form foci that give rise to an epidermis in culture [35]. The interfollicular epidermis reservoir is located in the upper part (the bulge) of the outer root sheath of the hair follicle [37] (see Figure 1). Basal keratinocytes from domestic mammals (dog [38, 39], cat [40], horse [41, 42], sheep [43, 44], rabbit [45], and chicken [46]) have also been isolated and propagated in culture. Chicken basal keratinocytes express p63, a keratinocyte stem cell marker found in mammals [46]. Interestingly, proliferative keratinocytes, comparable to basal-like keratinocytes can be derived from embryonic stem cells in human, mouse, chicken [26, 47, 48], or from induced pluripotent stem cells or iPSC, in horse [49].

Interfollicular dermal stem cells have also been identified in mouse and human skin [50]. These cells are important for dermis renewal and epidermal homeostasis regulation. It is important to note that these cells are multipotent cells and can differentiate into other cells of mesodermal and neural lineages [50].

After being hypothesized for a while, stem cells were also discovered in the hair follicle, predominantly in the bulge area [32] and in the dermal papilla [51]. There are two predominant types of stem cells in the bulge region: self-renewable cells and multipotent cells [52, 53] (for review see [54]). The former allows the renewal of the hair follicle and sebaceous gland, but also contribute to re-epithelialization of the epidermis during wound repair [55]. The latter are the hair follicles-associated pluripotent cells (HAP cells), which have the ability to differentiate into other cell types (i.e., nerve cells, glial cells, smooth cells) [56]. A characteristic of stem cells is that they remain in a prolonged quiescent state and are activated in response to hair renewal or skin injury. Different stem cells with have also been identified in the upper part of the hair follicle (isthmus and junction zone) [57]. Stem cells of the follicle hair bulge region have been identified in different mammalian species: dog [58–60], sheep [61], and pig [62]. In birds, epithelial stem cells are located in the collar bulge of the feather follicle above the dermal papilla [63]. In mammals, the bulge also harbors melanocytes stem cells [64], which in the human scalp have a shorter lifespan than hair stem cells, as they are programmed for around 20 hair cycles (D. Dhouailly, personal communication). Melanocyte stem cells serve as a reservoir for hair and skin melanocytes [6]. In birds, the melanocytes stem cells are located in an orthologous region of the feather follicle and serve only as reservoir for the feather melanocytes [7].

The dermal papilla, which is known to induce hair follicle [65], is located at the base of the hair follicle, and hosts mesenchymal stem cells. These cells are another source of iPSC [51]. Such cells were isolated in horse [66]. In chicken, comparable cells were also isolated in the dermal papilla of the feather follicle [67]. Interestingly, hair follicles constitute an important source of stem cells for regenerative medicine in mammals due to their easy accessibility. Moreover, basal keratinocytes and dermal fibroblasts are key cells for the development of skin equivalents.

## Tools and techniques of 3D skin culture—decades of innovations for human

### 3D versus 2D skin models

Numerous human skin models have been developed in the last 70 years, from skin explants cultivated ex vivo to in vitro reconstitution of skin equivalents or bioprinting techniques. One of the first mammalian skin model was developed by Medawar in 1948 [68]. It consisted of ex vivo skin cultures directly obtained from biopsies (rabbit, human). Quickly, in the 1950′s, these preliminary skin explant models were overshadowed by the advent and standardization of 2D cell culture methods. 2D models, are monolayer cells cultured on a solid surface under regular physico-chemical conditions. 3D models involve co-cultivation of the cells in three dimensions (in a spheroid or others volumes) in a way that they proliferate and interact with each other horizontally and vertically within an extracellular environment [69]. Despite its limits, the use of conventional 2D cell culture has prevailed for a long time to study different types of skin cells (keratinocytes, fibroblasts, melanocytes…) [1, 70]. It is only in the late 1970′s that 3D skin models saw a renewal thanks to Howard Green [36, 71]. 3D culture allows to obtain well differentiated cells and to co-cultivate different cell types together (e.g. keratinocytes associated with fibroblasts [72], with or without melanocytes [73, 74]). Moreover, it offers more possibilities than 2D culture by allowing to conduct more elaborate studies and in a wider range of applications (reviewed in [1, 75]). Indeed, with 3D models, various factors can be explored: (i) Environmental factors, such as spatial orientation and mechanical forces [76]; (ii) Physiological factors such as gradient of nutrients, gases (e.g., O_2_ with normoxia or hypoxia zones), or signalling molecules [1] and (iii) Interaction factors, such as cell-to-cell, cell-to-ECM, and cell-to-environment (like air) interactions [77]. In addition, 3D tissue cultures lead to material with an architecture close to real skin and provide better prediction of in vivo results. These models give also more accurate results for drug testing than 2D cell cultures, for which the results were increasingly questioned [78]. In addition, reconstructed skin turned out as an alternative to animal experimentation, notably for testing cosmetic products, transdermal drug delivery [79], toxicological assays [78], and UV effects [80]. Reconstructed skin has therefore emerged as a potent cost-effective, practical, and more ethical solution in response to the increasing demand for skin testing. In the last 30 years, extensive research has been conducted for human skin, from which numerous innovations and models have emerged. These models have various levels of complexity depending on the type of supports used, the number of cell types involved, and their positioning. The main human and mouse 3D skin models are listed in Table 1 and the principles to obtain some of them are described in the following paragraphs.

**Table 1:** **Major 3D skin models in humans and mice**

Type of models	Species	Specificities	References
Skin explants cultivated ex vivo (from biopsies)	Mouse	Skin explants cultured on chick chorioallantoic membrane (CAM)	[22]
Reconstructed human epidermis: on an acellular de-epidermized dermis (aDED) support	Human	Primary keratinocytes cultured onto an aDED to reconstruct an epidermis. The keratinocytes being differentiated through ALI	[81, 83]
Reconstructed mouse epidermis: on an acellular de-epidermized dermis (aDED) support	Mouse		[84]
Reconstructed human epidermis: on plastic filter support	Human	Keratinocytes cultured at ALI on an inert surface (e.g. polycarbonate insert filter)	[91, 92]
Reconstructed mouse epidermis: on plastic filter support	Mouse		[94]
Reconstructed human epidermis: on a dermal substitute made of biomaterials	Human	Seeding of keratinocytes on a dermal substitute made of biomaterials (collagen, hyaluronic acid, alginate…). A multilayered epidermis is obtained following the differentiation of keratinocytes at ALI	[89, 95]
Full thickness skin: on dermal substitute made of biomaterials and populated with fibroblasts	Human	Seeding of keratinocytes on a living dermal substitute (collagen mixed with fibroblasts). A multilayered skin equivalent is obtained following the differentiation of keratinocytes at the ALI	[99–101]
	Mouse	Mouse skin epidermis model. Mouse keratinocytes cultured at ALI on a living dermal substitute (type-I collagen with murine dermal fibroblasts)	[102]
Full thickness skin: DED populated with fibroblasts	Human	Keratinocytes seeded on a DED populated with fibroblasts and cultured at ALI	[97]
Full thickness skin: combining a DED populated with fibroblasts	Human	Keratinocytes seeded on a DED populated with fibroblasts and cultured at ALI	[98]
Complex Full thickness skin: pigmented models	Human	Skin equivalent additionated with melanocytes	[121]
Complex Full thickness skin: skins diseases related models	Human	Psoriasis or melanoma models	[123, 127]
Immunocompetent 3D skin equivalent	Human	3D skin model comprising of: dendritic cells (incl. Langerhans cells) or T-cells	[120, 123–125]
Full thickness skin: reconstituted from immortalized cells	Human	3D skin culture techniques using immortalized keratinocytes and fibroblasts to produce a multilayered human skin equivalent	[109, 110]
Full thickness skin: reconstituted from iPSCs-derived cells	Human	Multilayered epidermal structure reconstituted from human keratinocytes and fibroblasts cells derived from iPSCs	[113]
Full thickness hair-bearing skin: organoid system	Human	Multilayered epidermal structure exhibiting hairs reconstituted thanks to an organoid system	[117]
3D Skin obtained by bioprinting	Human	Automated process used to overlay sheets of cells to obtain a complex skin architecture	[129]
Skin-on-chip systems	Human	Technology combining reconstructed skin tissues with microsystems	[132]

### Reconstructed human epidermis (RHE)

**Figure 2:**
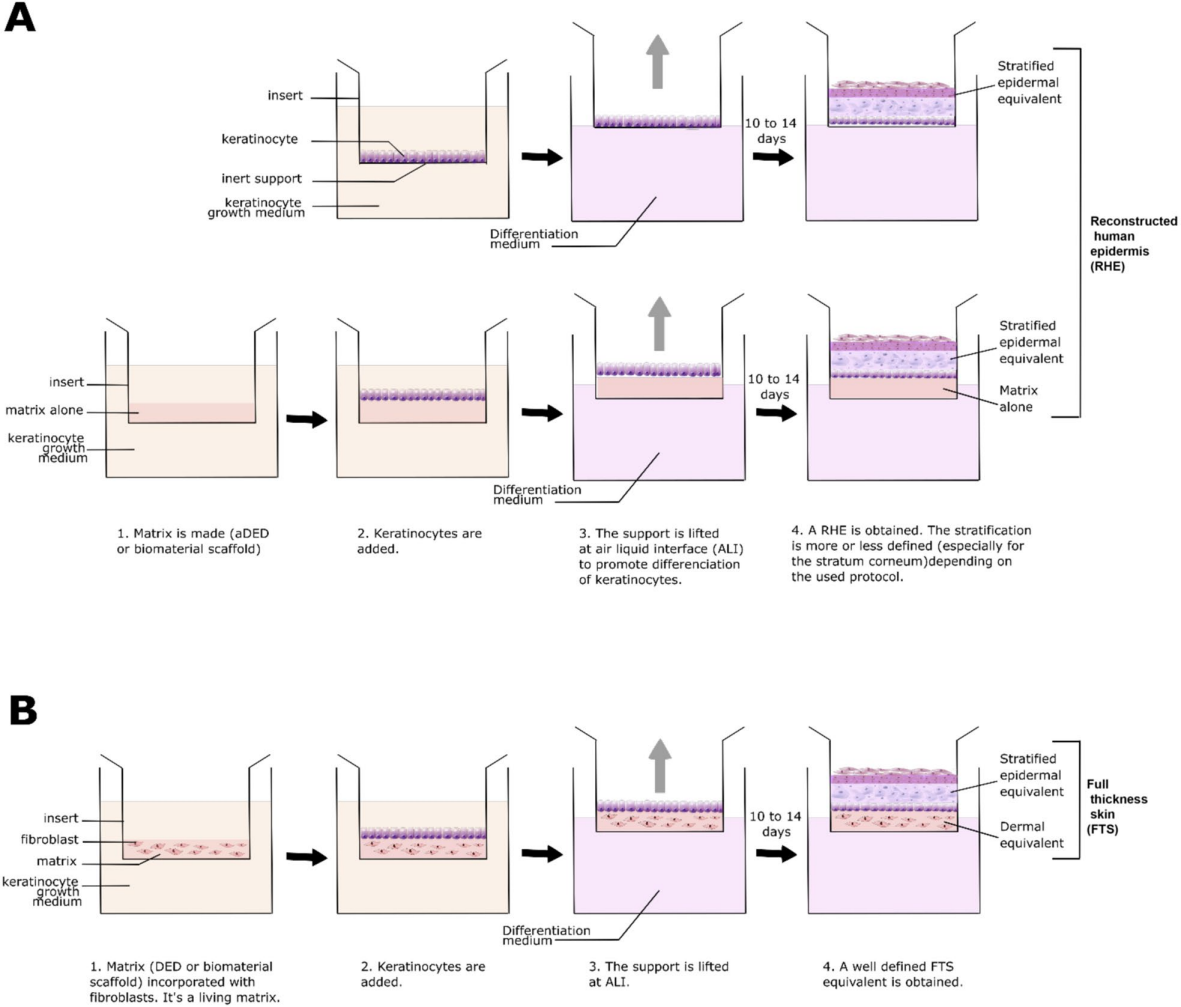
**Schematic representation of main 3D skin model techniques.** **A** To reconstruct human epidermis (RHE), keratinocytes can be seeded either onto an inert filter support or on a cell-free matrix/support. **B** To obtain human FTS with both a dermal and an epidermal equivalent, the matrix/support is populated with fibroblasts. In both models (**A**, **B**), the differentiation of the keratinocytes is promoted by cultivation at ALI. This step triggers the three-dimensional stratification of keratinocytes in multiple layers. In the most perfected models, the cornification process can be completed, resulting in skin or epidermis equivalents with all epidermis layers, including a well-defined cornified layer at their surface.

Human epidermis can be reconstructed (Figure 2) by cultivating keratinocytes on a support: (i) an acellular de-epidermized (aDED) matrix, (ii) inert filters, or (iii) a biomaterial structure forming a hydrogel or a lattice (Table 1). The aDED matrix was the first support utilized to produce a RHE exhibiting a cornified layer on its surface [81, 82]. To achieve this, primary keratinocytes isolated from human biopsies are seeded onto an aDED and allowed to differentiate for 2 weeks at the air–liquid interface (ALI) [81, 83]. Similar reconstructed epidermis (RE-aDED) are also developed for mouse [84]. Prunieras’ discoveries opened the way for the intensive development of in vitro epidermis reconstructive methods. Indeed, in most RHE models, primary keratinocytes are isolated from a donor and cultured as a monolayer to be amplified. Keratinocytes are next seeded onto a matrix support to allow their adhesion and proliferation. This support is subsequently lifted to the ALI to bring keratinocytes in contact with air. In about 2 weeks, the differentiation and stratification of keratinocytes results in a multilayered stratified epidermis similar to that found in vivo (reviewed in [85, 86]).

The second technique to produce RHE is a protocol using a support, a dermal substitute (DS), consisting of biomaterials only (e.g., collagen, alginate, hyaluronic acid, fibrin, etc.) (reviewed in [87]). The chosen biomaterial is often structurally similar to natural constituents of the extracellular matrix [88]. RHE-DS originated from the landmark study of Tinois [89], in which the support synthesis implies the crosslinking of collagens layers (with human collagen IV on the top). Collagen IV, the major in vivo component of the basal membrane, appeared to be a great substrate for keratinocytes, favoring both cell proliferation and anchoring to the dermal substitute support. With this method, a well-organized multilayered RHE with a structured basement membrane is obtained. This model was standardized, scaled up, and produced at factory level, leading to EpiSkin™ product (L’Oréal), one of the first reconstructed skin validated for in vitro corrosivity testing and irritation testing of chemicals (reviewed in [90]). This model has taken a step forward in reducing animal testing for chemicals and legitimated the high potential of reconstructed 3D skin models.

Culturing keratinocytes onto acellular inert filter supports is another alternative to produce RHE. Polycarbonates filters were first tested by Rosdy et al. [91]. With this support, a well differentiated and multilayered skin RHE and reconstructed mouse epidermis RME) were obtained, similar histologically to real skin [91–94]. This method was at the origin of a skin production factory, supplying a novel reconstructed skin (SkinEthic™), initially for testing cosmetics and later for testing chemical corrosion and irritation [93].

RHE models are commonly used to evaluate cosmetics or topical drug products for irritation, phototoxicity (response to UV-lights) [95], corrosion, or skin sensitivity (due to immune reaction) [96]. Permeation of the skin and efficacy of cosmetic products are also determined on RHE skin models [92].

### Full-thickness skin (FTS) models

Going one step further, human FTS models can be produced using a living dermal equivalent populated with fibroblasts (Fig. 2). As such, the reconstituted 3D skin possesses two compartments: one dermal compartment with fibroblasts and one epidermal compartment with keratinocytes. The living dermal substitute can be produced either on (i) a DED matrix [97, 98] or (ii) a biomaterial scaffold support, in which fibroblasts are incorporated. Referring to the method of Bell [99, 100], human and mouse FTS can be produced using a fibroblast-populated collagen lattice [101, 102]. The collagen scaffold retracts when submerged in a defined medium forming a solid lattice. The integrated fibroblasts are, in the Bell’s model, restricted to the retracted biomaterial [103]. The matrix contraction occurs due to physical forces exerted by incorporated fibroblasts [104, 105]. A variety of biomaterial has also been used in combination with fibroblasts to produce FTS [106]. These 3D scaffold matrices are presumed to be similar to the real dermal tissue at functional, structural, and mechanical levels [1, 107]. Moreover, the proliferation of fibroblasts is, in fact, inhibited in the dermal equivalent due to retrocontrol mechanisms and to biochemical confinement [103, 108].

FTS can be reconstructed with primary cells as described above, but also with immortalized cells or skin cells derived from stem cells (iPSC or others). Indeed, one major issue was to reconstitute 3D skin independently of skin donors and primary cells. Physiologically relevant FTS was obtained using human TERT-immortalized keratinocytes and fibroblasts (TERT-HSE) [109]. Reconstruction of well-differentiated skin was also established using immortalized primary human foreskin keratinocytes [110]. Pioneer studies demonstrated that human and mouse keratinocytes obtained from embryonic stem cells were usable for full reconstruction of pluristratified epidermis [47, 48]. More recent studies have shown that iPSC can be differentiated into keratinocytes [111, 112], fibroblasts [113], or melanocytes [114]. Basal membrane obtained with iPSCs-derived fibroblasts were proven to exhibit similar characteristics to primary fibroblasts (review in [115, 116]). Itoh et al*.* obtained for the first time a well differentiated full-thickness 3D skin equivalents using exclusively human iPSC-derived keratinocytes and fibroblasts [113]. Recently, hair-bearing human skin was reconstructed by using human pluripotent stem cells only, thanks to an organoid system [117].

### Complexification of 3D skin models: toward fully functional skins

Current challenges are now focusing on engineering even more physiologically relevant skin models (reviewed in [118]). This complexification can be made by incorporating different types of skin components such as vasculature [119], appendages (hair follicles, glands), pigmentation, innervation, immune cells [120], or hypodermis. Reconstructed human pigmented epidermis (RHPE) possessing melanocytes cells is available [121] and commercialized (e.g., SkinEthic RHPE™, MelanoDerm™). Many other improvements have been obtained by incorporating other cell types. For example, fat sub-cutaneous hypodermis was reproduced by adding adipocytes [122], and reconstructed immune-competent skin was obtained (see Table 2) following the addition of T-cells [123], Langerhans cells [124, 125], or mDCs [120]. The development of skin disease models is also the object of many efforts (reviewed in [126]), like in vitro skin cancer models (constituted with melanoma cells and/or melanocytes [127]) or psoriasis models [123]. These models often required optimized immuno-competent 3D skin due to the complex cross-talk involved between various immune cell types and cytokines [128].

The elaboration of the next generation of 3D skin models benefits from the development of more advanced technologies. For example, bioprinting is one promising solution that had emerged over the last decade. Skin reconstitution by bio-fabrication can be made according to three techniques: (i) Inkjet-based, (ii) pressure-assisted, or (iii) laser-assisted bioprinting (reviewed in [129]). Bioprinting is an automated approach consisting in the deposition of cells and biomaterials (e.g., bio-inked hydrogels) so as to imitate very closely the real skin structure [130]. This technique is highly reproducible and allows a precise positioning of the cells. In the past years, a broad range of biomaterials has been utilized as “bio-ink” to produce full thickness skins: natural polymers including collagen, gelatin, alginate, and synthetics polymers including polyethylene glycol and poly lactic-co-glycolic acid [130]. The expansion of bioprinting skin manufacturing has rendered possible the production of very complex 3D skin exhibiting, for example, both vasculature and lymphatic capillaries [131]. The last improvement in these models is the development of skin-on-chip models, a technology combining cell/tissue with microsystems (see review of [132]).

## 3D skin models for livestock, poultry and companion animals: current and future

There is a huge gap between 3D skin research for human and for veterinary purposes, both in abundance and in complexity. Although skin models were first conceptualized in animals, animal skin models are no longer considered relevant for use in the pharmaceutical and cosmetic industry since the development of human 3D skin models. As a result, the number of animal 3D skin models available nowadays is very limited [133]. These models are either old models developed for human purposes (notably explants) or novel models developed for veterinary medicine or comparative biology. Nowadays, animal skin model development is focusing on animal companion species known to have predispositions for skins diseases, like dogs. The available 3D skin models related to livestock (pig, cow, sheep, rabbit), poultry (chicken), and companion animals (dog, horse) are listed in Table 2.

### Skin explants in animals

Skin explants were reported in rabbit, pig, cow, and dog as referenced in Table 2. Initial skin explants were developed in the 1940s and abandoned in favor of monolayer cell cultures. A resurgence of interest appeared with Kondo’s study using rabbit ear skin biopsy as split thickness skin explant which contains an epidermis with an upper dermis portion. This model can be cultured stably up to 12 weeks in a diffusion chamber [134]. It opened the way to skin explants in other farm animals such as sheep, cow, or pig. Epidermis explant from bovine hoof was cultured at the ALI and remained viable in short-term culture [135]. Interdigital skin explant from ovine hoof cultivated in anaerobic conditions was viable during 72 h [136]. Pig skin initially elicited the most interest as human skin model until the availability of human RHE and FTS [133]. Domestic pig skin was an interesting model due to its similarity with human skin in term of lipids composition and permeability [137–139] and the low amount of hair [140]. Pig skin biopsies were used for testing drug/products either (i) directly on freshly collected skins or (ii) after a time of culture of the skin for 2 weeks. Protocols using skin collected from other parts of the pigs’ body or shaving down the dermis so as to render skin thickness more representative of human skin (e.g. [141]) have been reported. For chicken, skin explants and skin culture from chick embryos have been used. Chicken skin explants can be cultivated at the ALI on a grid or an insert [142], on semi-solid agar, or grafted on a chorioallantoic membrane (CAM) [22]. Note that the grafted method on CAM is also feasible with human or mouse explants [22, 143] (Table 1). Chicken skin explants were frequently used in the past to study skin appendage development (for e.g. [22, 144]). Two canine skins explant models which could be maintained viable for 2 weeks were recently published [133, 145].

**Table 2:** **3D Skin models in domestic animals/rent and companions mammals and birds**

Type of models	Species	Specificities	Potential applications	References
Skin explants from biopsies cultured ex vivo	Rabbit	Skin ears epidermis organotypically cultured ex vivo in a diffusion chamber	Optimisation of explant culture conditions. Skin keratinization model	[134]
	Pig	Ears skin cultivated ex vivo for dermatological pharmacokinetic purposes	Tool for topical application of pharmaceutic products	[137]
	Pig	Skin explants obtained from Gottingen minipig cultured in medium. Dermis thickness shaved down from 20 to 2–3 mm	Surrogate for human skin for skin safety studies	[141]
	Pig	Pig ear skin explant seeded onto a gelatin coated polycarbonate transwell insert and cultured ex vivo at ALI	Tool to assess UV effects, apoptosis or to evaluate the photoprotective capacity of cosmetic formulations	[138]
	Pig	Dorsal skin punches biopsied cultured ex vivo as explants (with a cornified layer) and maintained at ALI	Optimization of animal explants cultures methods. May be a tool to study animals skins diseases	[133]
	Cow	Bovine claw epidermis explants. Hard horn removed and the remaining tissue constituted by a dermis and an epidermis (with a cornified layer) was cultivated in medium at ALI on a metal grid	Investigation of epidermal keratinization	[135]
	Sheep	Interdigital skin explants cultivated ex vivo	Investigation of anaerobic bacterial infection in the skin	[136]
	Dog	Skin punch biopsies cultured ex vivo 2 weeks immersed in serum-medium	Study of dog skin physiology or pharmacological responses	[145]
	Dog	Lateral flank/abdominal skin punches. Cornified layer maintained in ALI during culture	Optimization of animal explants cultures methods. May be a tool to study animals skins diseases	[133]
	Chicken	Skin explants cultured on chick chorioallantoic membrane (CAM)	Development studies on chicken skin appendages	[22]
	Chicken	Embryonic skin explants, cultivated at ALI on a filter based or semi-solid agar	Development studies on chicken skin appendages, Simplified model to study responses underlying skin burning	[142]
3-D reconstructed epidermis with primary cells	Dog	Epidermis equivalent obtained from primary keratinocytes cultured at ALI onto a porcine aDED matrix	Characterization of a canine epidermis model. May be a tool for dermatological researches	[39]
	Horse	Epidermal-like architecture obtained from culture of keratinocytes on an inert transwell system	Tool for modelling skins diseases, drug testing and maybe equine regenerative medicine	[41]
3-D reconstructed FTS with primary keratinocytes cells (modified Bell’s method)	Sheep	Skin equivalent obtained from culture at ALI of primary ovine keratinocytes onto a dermal (primary ovine fibroblasts) collagen matrix. Used for 3D induction of follicles with the incorporation of the whole derma papillae or cultured papilla cells into the skin equivalent	Study of chemical that may facilitate hair follicle development	[44]
	Sheep	Skin equivalent obtained from primary lamb keratinocytes (PLKs) culture at ALI 12 days on a living collagen scaffold (type I rat tail) with 3T3 J2 fibroblasts	Model of infection in the skin	[43]
	Dog	Skin equivalent obtained from primary canine keratinocytes cultured at ALI onto a dermal (canine primary fibroblasts)-collagen (type I rat tail) lattice	Investigate canine skin biology and pathology	[38]
	Horse	Skin equivalent obtained from the seeding of primary horse keratinocytes onto a type-I collagen dermal scaffold as matrix and cultured 2 weeks at ALI	Characterization of equine skin equivalent, may be further a tool for equine regenerative medicine	[42]
3-D reconstructed epidermis with keratinocytes derived from progenitor cells	Dog	Reconstruction of a canine epidermis with a stratum corneum. Canine progenitor epidermal keratinocytes (CPEK) seeded on a living dermal substitute (primary fibroblasts mixed with collagen) and cultured at ALI	Tool to study *stratum* *corneum* canine barrier function	[147]
	Dog	Epidermal equivalent obtained from canine progenitor epidermal keratinocytes (CPEK) cultured at ALI onto a type-I collagen dermis equivalent	In vitro tool to study tight junctions in dogs	[148]
3-D reconstructed epidermis with keratinocytes derived from iPSC	Horse	Conditional reprogramming of cells. Culture of keratinocytes derived from equine induced pluripotent stem cells (eiPSCs) at ALI	Development of equine skins models using conditional reprogramming. Eventual utility for autologous transplantation	[149]
3-D reconstructed epidermis with bulge cells-enriched keratinocytes	Dog	Use of bulge stem cells keratinocytes from canine hair follicles. Cultured at ALI on a collagen-canine fibroblasts lattice	May be a model for dog skin regenerative therapy	[150]

### Skin equivalents in animals

To date, fewer than a dozen skin equivalent 3D cultures have been published for domestic animals, and only for dog, horse, and sheep (Table 2). In these models, methods developed with human skin were adapted to veterinary species. Reconstructed epidermis was obtained for dog and horse. For that, primary isolated keratinocytes were cultured onto: (i) pig acellular DED matrix [39], or (ii) an inert transwell matrix [41]. Well-differentiated FTS equivalents were also obtain with a modification of the Bell’s method (see above paragraph 2.3) for four species: ovine [43, 44], canine [38, 146], feline [40], and equine [42]. Of note, Watson incorporated efficiently whole derma papilla or cultured papilla cells into reconstructed ovine skin [44]. Other protocols utilized long-term progenitor epidermal cells lines. Canine 3D organotypic skin models were obtained using canine progenitor epidermal keratinocytes onto an acellular collagen gel layer. Two studies based on this protocol produced canine epidermal equivalent with a well-defined cornified layer [147, 148]. Keratinocytes derived iPSC [149] and bulge cells-enriched keratinocytes from dog hair-follicles [150] have also been used in reconstructed equine and canine epidermis, respectively. As of today, there are no skin equivalents for birds.

### The future of 3D skin models in veterinary medicine

As mentioned above, the availability of 3D animal skin models for veterinary purposes (health, well-being, research) is very limited. With the remarkable development of human skin equivalents, animal skin models are no longer models for humans, but may benefit from human skin model developments.

The animal 3D skin models are still important for comparative biology and for several skin diseases, notably dermatitis (e.g., *Staphylococcus* *aureus* skin infection and atopic dermatitis in dog). In different species, such models could be used to investigate the effects of microbiota on skin permeability. In the vaccinology field, they may become more important in the future to study transcutaneous delivery of antigens for vaccination, a new promising mode of vaccine administration to animals. Such skin models may also help develop molecule administration through the skin for a general effect. Several antiparasitic molecules are already delivered by such a way (“spot on” administration) with great success (e.g., Fipronil against fleas and ticks in cats and dogs). Lastly, these animal models could allow to evaluate the impact of toxic products for animal skin, notably for animals bred for human consumption and for food products containing skin components.

Skin 3D models were previously shown to be valuable models to study virus/skin interactions, notably with herpesviruses (e.g., Varicella Zoster Virus and Herpes Simplex Virus 1) [151, 152]. Our research interests focus on the Marek’s disease virus, an oncogenic avian herpesvirus that is persistently shed into the environment by infected chickens, whether vaccinated or not. This virus is produced by the epidermal cells of the feather follicles wall. The feather follicle epithelium is the only tissue capable of producing tremendous amounts of infectious mature virions. The ultimate goal in poultry production is to stop virus shedding in skin danders in order to eradicate the disease. Compared to live animal experimentation, using 3D chicken skin models would be useful as they allow various manipulations (e.g., addition of drugs, gene overexpression, gene knock-in or knock-out) do not necessitate an animal facility, and are more acceptable in term of ethics. Therefore, a reconstituted chicken epidermis or FTS model would offer a better opportunity to identify cellular and viral molecular determinants involved in virions shedding and to develop new strategies to counteract it.

## Conclusion

The available protocols for 3D skin in human are plethoric and still expanding. They are already used for many applications in cosmetology and toxicology and are regularly adapted to develop new in vitro models of skin diseases. Importantly, these models represent great alternatives to animal experiments for skin testing. Although the number of such protocols are still limited in domestic animals, their feasibility with methods commonly used in humans was recently demonstrated in a few veterinary species. The development of such new models may greatly help the study of skin diseases as well as the examination of transcutaneous delivery of pharmaceutical molecules and vaccine antigens.

## Data Availability

Not applicable.

## Abbreviations

aDED: Acellular de-epidermized dermis
ALI: Air–liquid interface
DED: De-epidermized dermis
DS: Dermal substitute
FTS: Full-thickness skin
iPSC: Inducible pluripotent stem cells
RHE: Reconstructed human epidermis
UV: Ultraviolet
2D: Two-dimensional
3D: Three-dimensional
